# Kinome and phosphoproteome of high-grade meningiomas reveal AKAP12 as a central regulator of aggressiveness and its possible role in progression

**DOI:** 10.1038/s41598-018-19308-y

**Published:** 2018-02-01

**Authors:** Carolina Angelica Parada, Joshua Osbun, Sumanpreet Kaur, Youssef Yakkioui, Min Shi, Catherine Pan, Tina Busald, Yigit Karasozen, Luis Francisco Gonzalez-Cuyar, Robert Rostomily, Jing Zhang, Manuel Ferreira

**Affiliations:** 1 Departments of Neurosurgery/University of Washington School of Medicine, University of Washington Medical Center, Seattle/WA, 98195 USA; 2 Department of Pathology/University of Washington School of Medicine, Harborview Medical Center, Seattle/WA, 98104 USA

## Abstract

There is a need to better understand meningioma oncogenesis for biomarker discovery and development of targeted therapies. Histological or genetic criteria do not accurately predict aggressiveness. Post-translational studies in meningioma progression are lacking. In the present work, we introduce a combination of mass spectrometry-based phosphoproteomics and peptide array kinomics to profile atypical and anaplastic (high-grade) meningiomas. In the discovery set of fresh-frozen tissue specimens (14), the A-kinase anchor protein 12 (AKAP12) protein was found downregulated across the grades. AKAP12 knockdown in benign meningioma cells SF4433 increases proliferation, cell cycle, migration, invasion, and confers an anaplastic profile. Differentially regulated pathways were characteristic of high-grade meningiomas. Low AKAP12 expression in a larger cohort of patients (75) characterized tumor invasiveness, recurrence, and progression, indicating its potential as a prognostic biomarker. These results demonstrate AKAP12 as a central regulator of meningioma aggressiveness with a possible role in progression.

## Introduction

Meningiomas account for 30% of reported brain tumors^1^. The World Health Organization (WHO) histologically classifies tumors into three grades: benign (I), atypical (II) and anaplastic (III)^2^. Although most meningiomas are benign, 15–35% represents atypical or anaplastic forms^3^. High-grade meningiomas (II and III) do not respond well to surgery and lead to decreased survival. Radiation is used as adjuvant to treat progressive tumors since there are no efficient chemotherapies for the management of high-grade tumors that fail treatments. Understanding pathways of oncogenesis that drive high-grade meningiomas is important to improve current diagnosis and treatment. Genetic abnormalities have fallen short as predictive biomarkers. Monosomy 22 or alterations in *NF2* are present in more than half of meningiomas^4,5^. Although genomic alterations increase with grade, they do not predict outcomes, stage or history. Over 40% of atypical meningiomas will reoccur after gross resection. Biomarkers for identification of these histologically identical WHO II tumors do not exist. Anaplastic meningiomas lead to fatal outcomes despite surgery, radiation, and experimental medications. Recent genomic discoveries^6,7^ have not led to improvement in therapies. The mechanism behind malignancy and tumor genesis is unknown, limiting development of alternative therapies.

The functional regulation of cells is a complex and dynamic process. Genetic alterations can drive the activity state of pathogenesis, but the interaction between the transcriptome and the proteome can interact in a feedback or feed forward fashion. Phosphoproteomic techniques have been widely employed to identify kinase activity, which may lend itself to pharmaceutical blockade. There have been few proteomic investigations focused on high-grade meningiomas, most of them gel-based approaches^8–11^ which have limitations in resolution, reproducibility^12^, poor representation of minimally expressed proteins, difficulty with highly acidic/basic proteins, and those with extreme size or hydrophobicity^13^. The majority of these studies have been limited to protein expression^8–11,14,15^ overlooking post- translational modifications, with no cell-based functional characterization^8–11,14,15^,which relate to function and malignancy of the disease.

To map the mechanisms of aggressiveness we aimed to investigate changes in protein phosphorylation across WHO grades of human meningiomas. We utilized two high-throughput techniques: the unbiased iTRAQ LC MS/MS and the biased Pamchip® peptide array. This strategy was used to better understand the underlying mechanisms of meningioma malignancy, aid in the development of novel therapies and markers of poor prognosis. Here we report the kinomic and phosphoproteomic signatures of high-grade meningiomas, the biomarker potential and regulatory role of AKAP12 in meningioma malignancy.

## Results

### Sequencing for *SMO*, *KLF4*, *TRAF7*, *NF2*, and *AKT* E17K by Molecular Inversion Probes (MIP)

We performed targeted sequencing of *SMO*, *KLF4*, *TRAF7*, *NF2*, along with the screening of *AKT1* E17K in a set of 320 individual tumors. The screening was also performed in a discovery set of fourteen well-characterized individual human meningioma tissue samples (I:5, II:5, III:4) (Supplementary Table S1), and four benign human meningioma cell lines (HBL-52, Ben-Men-1, SF4433, and SF4068), one atypical (SF6717), two anaplastics (SF3061 and KT21-MG1), and unknown WHO grade, CH157-MN. We did not find correlations between genotype and grade or long-term outcomes in the cohort of 320 samples (unpublished observations), discovery set (14 samples) or cell lines (Supplementary Table S2).

### iTRAQ profiling of high-grade meningiomas reveals specific phosphoproteomes and kinase activity

**Figure 1:**
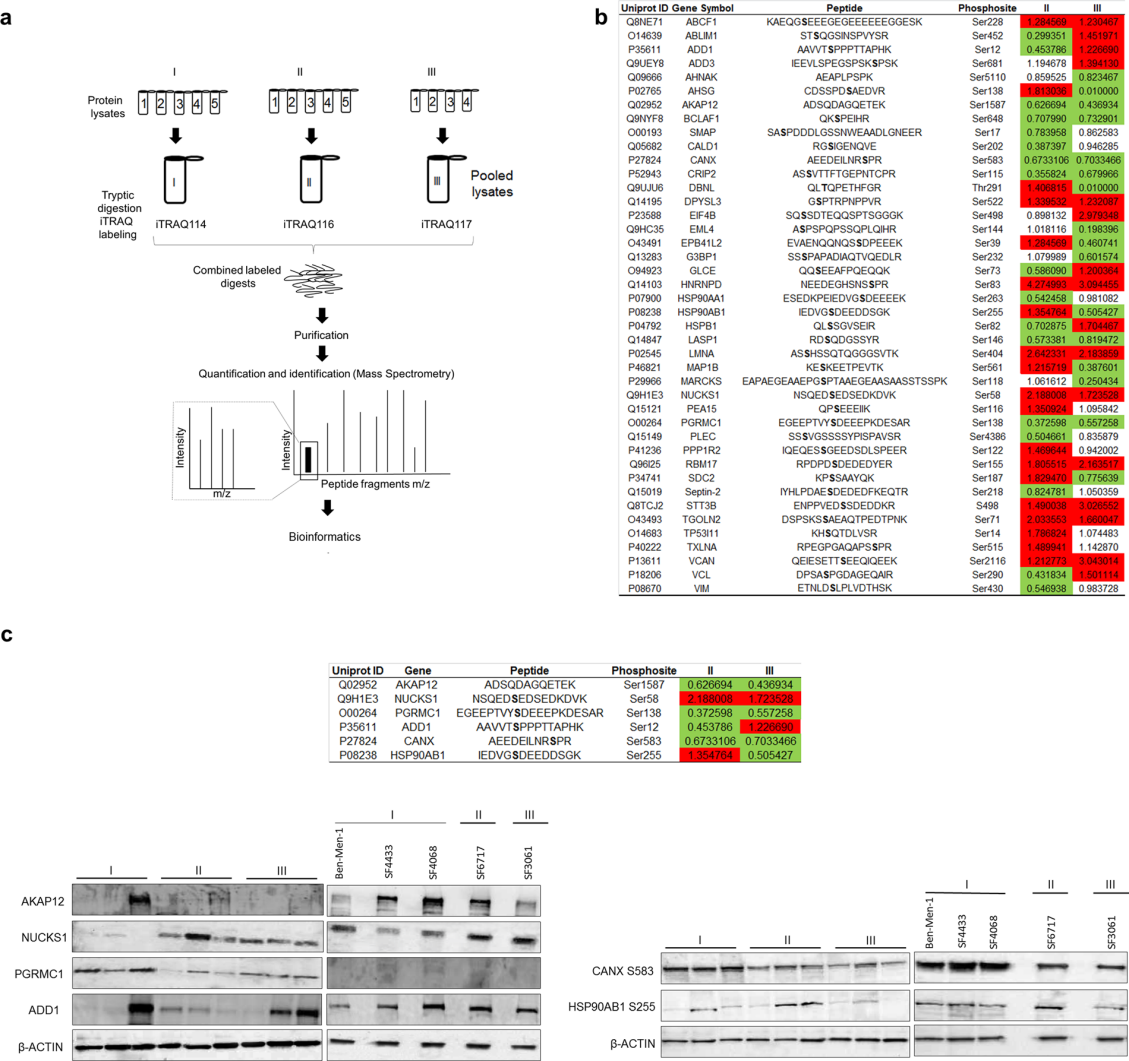
Meningioma iTRAQ LC MS/MS and Western Blot validation. (**a**) iTRAQ LC MS/MS workflow applied to meningioma fresh-frozen tissue samples. (**b**) List of 42 differentially phosphorylated proteins in grade II or III meningiomas versus the benign group. (**c**) Western blot (cropped lanes) of different grades of individual meningioma fresh-frozen tissue and cell line protein extracts to validate the iTRAQ-based quantification of six selected candidate proteins, including AKAP12, NUCKS1, PGRMC1, ADD1, CANX Ser583, and HSP90AB1 Ser255. Red: hyperphosphorylation; Green: hypophosphorylation. See Supplementary Figure S2 for western blot quantification.

iTRAQ LC MS/MS was performed on the fourteen meningioma fresh-frozen tissue specimens previously genotyped. The iTRAQ LC MS/MS workflow is shown (Fig. 1a). A total of 649 unique phosphopeptides from 165 proteins were identified (Supplementary Table S3). Forty-two peptides were altered (fold change > 20%) (Fig. 1b). The Gene Ontology (GO) and Principal Component Analysis (PCA) are shown in Supplementary Figure S1. According to protein class, 28.6% (n = 12) of phosphopeptides belong to cytoskeleton proteins. Classified by molecular function, these proteins are related to binding and structural activity (45.2%, n = 19; 28.6%, n = 12, respectively). Seventeen (40.5%) phosphoproteins were involved in metabolic processes. The cellular component analysis identified 31.0% (n = 13) of phosphoproteins located in cell parts other than extracellular matrix, extracellular region, macromolecular complex or organelles. Among the 42 differentially regulated phosphopeptides, 36/42 and 32/42 were altered in Grade II and III, respectively. For validation, we studied six proteins with unique patterns. The proteins selected for validation were selected based on their fold changes/patterns, and significant changes on protein level without considering multiple testing, corrections, or biological relevance. This is a commonly employed strategy in discovery proteomics to address proteomics-specific issues like small effects due iTRAQ ratio compression, and few replicates due to reagent high cost, instrument time availability and others^16^. AKAP12 Ser1587 was inversely proportional to tumor grade (0.63 and 0.44 fold, respectively) and showed the greatest significance (p = 0.000039 III:I); NUCKS1 Ser58 was upregulated in II and III (2.2 and 1.7 fold, respectively); PGRMC1 Ser138 was downregulated in both (0.37 versus 0.56 fold, respectively); ADD1 Ser12 was downregulated in grade II and upregulated in III (0.45 and 1.2 respectively); CANX Ser583 is downregulated across the grades (0.67 and 0.70 fold changes in grades II and III compared with grade I, respectively); HSP90AB1 Ser255 was upregulated in grades II and downregulated in III (1.35 and 0.50 fold, respectively); When site specific phospho-antibodies were not available for the selected candidates, we chose to validate how changes in detection of total protein expression correlated with phosphorylation. We studied three individual tissue samples from each of the three pooled samples (grades I, II, III). Except for ADD1 which levels of total protein did not show significance (p > 0.05), we found marked consistency between changes in phosphopeptide abundance and protein level (Fig. 1c, Supplementary Figure S2), suggesting that the differential detection of phosphopeptides may have been in part due to changes in protein levels across grades. We then determined whether these grade-dependent patterns of expression were recapitulated in meningioma cell lines from different grades. The patterns of expression in cell lines were mostly consistent with those found in patient samples (Fig. 1c, Supplementary Figure S2), supporting the relevance of the cell lines as models for functional studies.

### AKAP12 knockdown leads to migration, invasion, proliferation and cell cycle progression

**Figure 2:**
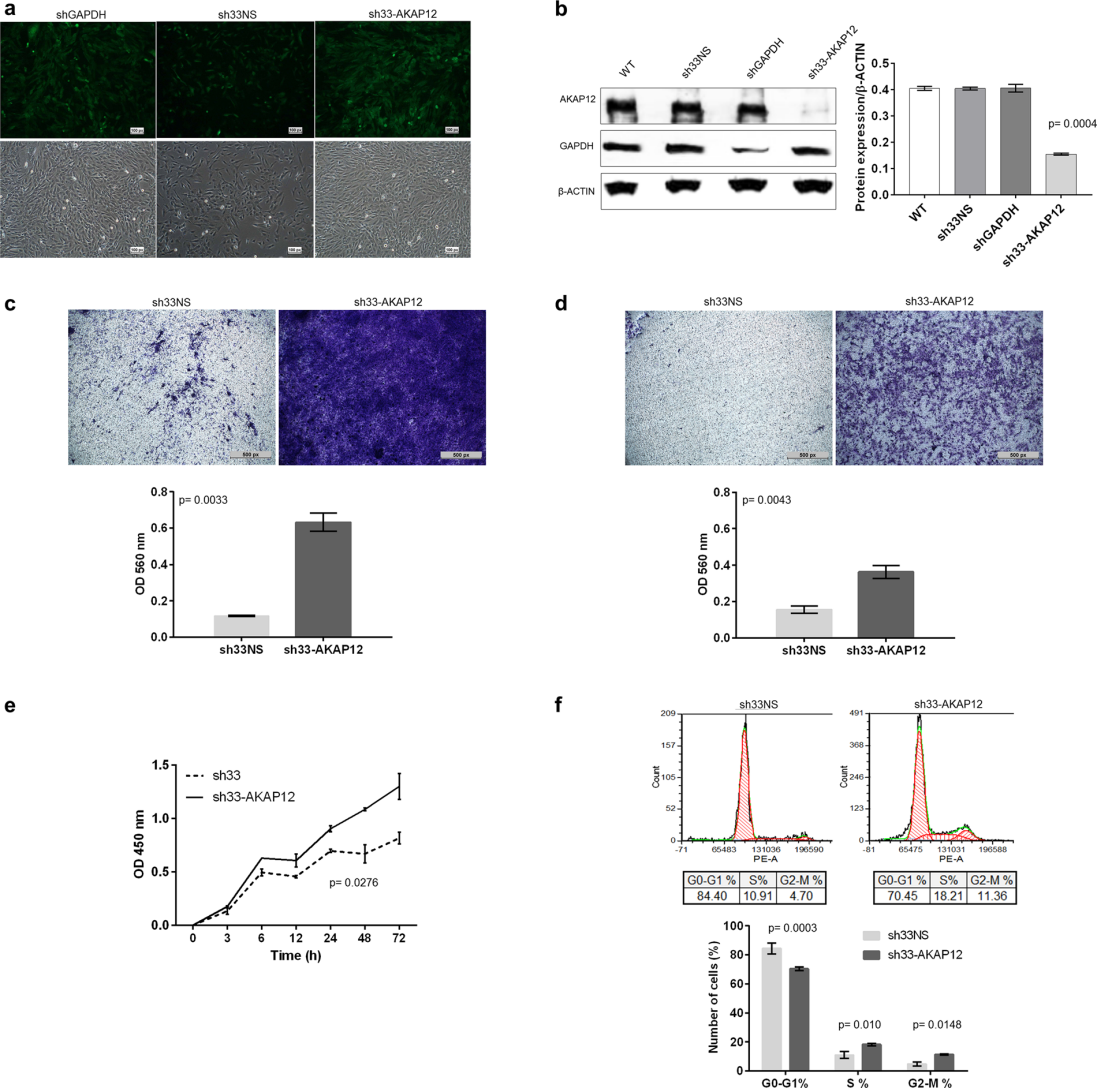
AKAP12 knockdown and functional assays. (**a**) GFP^+^ cells sorted by FACS after transfection with pGIPZ GAPDH shRNA (shGAPDH) – positive control, pGIPZ Non-Silencing shRNA (sh33NS) – negative control, and AKAP12 shRNA (sh33-AKAP12), (original, magnification 10x). (**b**) Gene knockdown efficiency was quantified by Western Blotting (cropped images) (p = 0.0004). Proteins levels were quantitated by densitometry using Image Studio Lite Version 5.0. Graph represents AKAP12 expression normalized to β-ACTIN (Y axis) versus cell line protein extracts (X axis). (**c**) Migration assay after 48 hours**:** sh33-AKAP12 versus sh33NS (p = 0.0033) (original, magnification 10x). (**d**) Invasion assay after 48 hours**:** sh33-AKAP12 versus sh33NS (p = 0.0043) (original, magnification 10x). (**e**) sh33-AKAP12 versus sh33NS proliferation curve (0–72 hours) measured by BrdU incorporation (p = 0.0276).(**f**) Cell cycle distribution of sh33-AKAP12 versus sh33NS assayed by determining cellular DNA content (G0-G1%, p = 0.0003), (S %, p = 0.010), (G2-M %, p = 0.0148). P-values were calculated using Student’s t test. Error bars denote ± SD.

Since AKAP12 expression has been found downregulated in other cancers^17–19^, and a clear correlation was established between AKAP12 expression and grade, we chose to investigate the effects of AKAP12 silencing on a benign phenotype using the SF4433 cell line which presented robust expression of AKAP12 (Fig. 1c, Supplemental Fig. S2). After transfection, GFP^+^ cells with AKAP12 shRNA (sh33-AKAP12), or either shGAPDH (positive control), and non-targeting negative control (sh33NS) were sorted (Fig. 2a). Compared to controls, protein levels were decreased approximately 85% by AKAP12 silencing (sh33-AKAP12) (p = 0.0004) (Fig. 2b). The AKAP12 knockdown correlated with enhanced migration (~6-fold; p = 0.0033) and invasion (~3-fold; p = 0.0043) (Fig. 2c,d). AKAP12 downregulation also increased proliferation by 1.7 fold after 72 hours (p = 0.0276; Fig. 2e). Consistent with increased cell growth, cell cycle distribution demonstrated low percentages of sh33-AKAP12 cells in G0-G1 and high in S and G2-M (Fig. 2f). Collectively, these data support a role for AKAP12 in suppressing invasive and proliferative phenotypes associated with meningioma malignancy.

### AKAP12 expression inversely correlate with kinase activity

**Figure 3:**
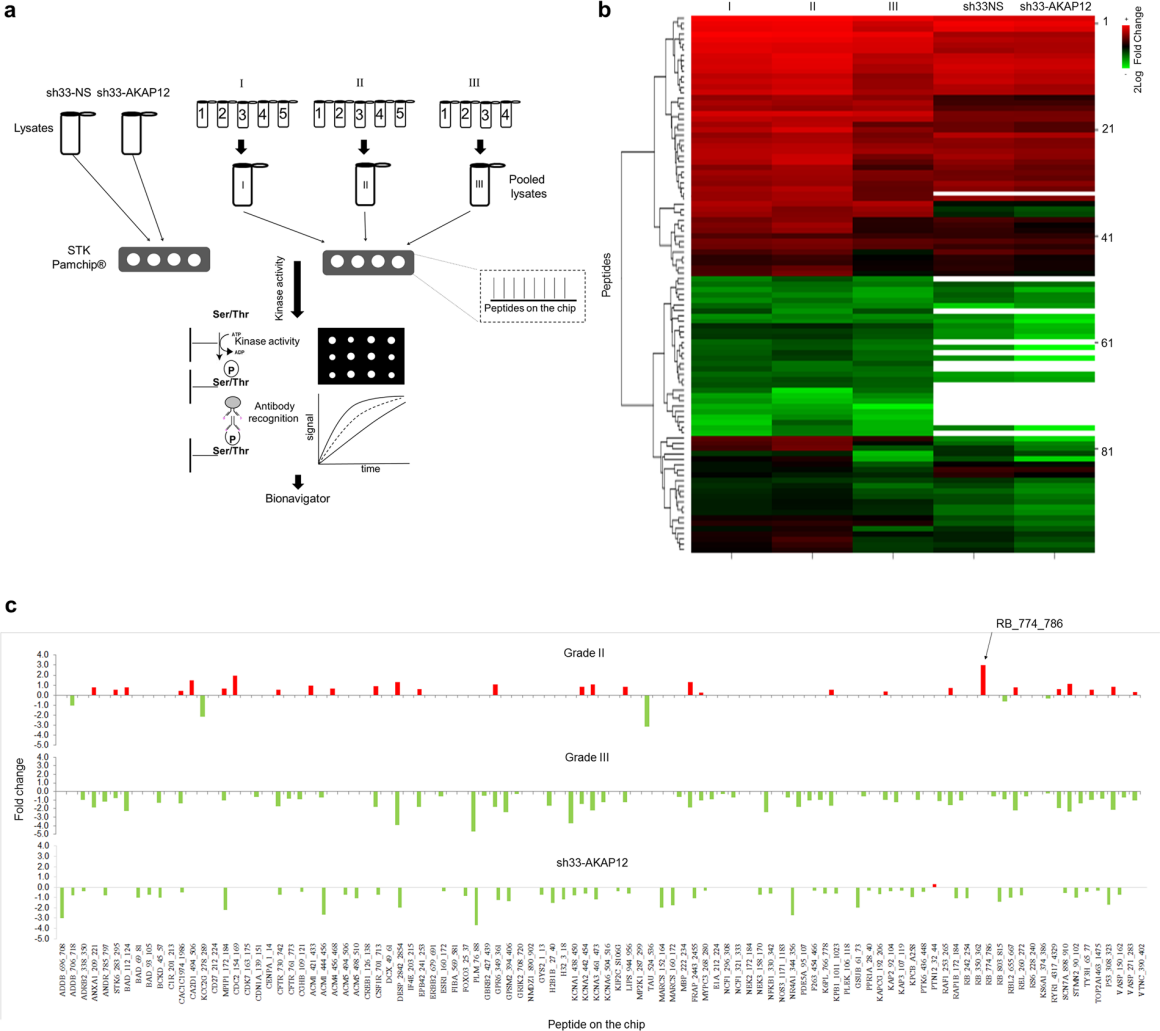
Serine/threonine kinomic profiling of meningioma fresh-frozen tissue and AKAP12 gene knockdown. (**a**) STK chip array workflow applied to meningioma fresh-frozen tissue and cell lines. (**b**) Heat map showing 2Log transformed signal intensity of differentially phosphorylated peptides (Y axis) in each sample (X axis) submitted to the STK chip array. The analysis detected 100/140 phosphorylated peptides above limit of detection in meningioma tissue extracts and 86/140 in cell lines lysates. Blanks indicate fourteen not detected in the cell line protein extracts **c:** Bar graph representing 2Log fold changes. Phosphopeptides with p > 0.05 and 2Log fold changes > 10% were considered significant (Y axis). Significant phosphorylated peptides are shown in the X axis. sh33-AKAP12 versus sh33NS identified 58/100 significant peptides. Grade II and III meningioma versus grade I showed 34/100 and 60/100 differentially phosphorylated peptides, respectively. Hyperphosphorylated peptides are indicated in Red. Hypophosphorylated peptides are represented in Green. P-values by ANOVA or Student’s t-test as appropriate.

We sought to determine whether the increased malignancy and decreased expression of AKAP12 in high-grade meningiomas and sh33-AKAP12 cells were connected through kinase regulation. We identified no evidence of tyrosine phosphorylation probably because phosphorylation on Ser/Thr is more common^20–22^. Since phosphorylated Ser/Thr were found abundant, we pursued kinome profiling of pooled I, II and III meningiomas, the sh33-AKAP12 and sh33NS cells using the PamChip® Ser/Thr kinase (STK) peptide array (Fig. 3a). Signal intensities of peptides above the limit of detection were clustered and represented as a heat map (Fig. 3b, Supplementary Table S4). Kinase activity was detected in 86/100 peptides between the AKAP12 control and knockdown cell line lysates, and 100/100 peptides in WHO II and III pooled extracts. For the fold change analysis, only peptides with p < 0.05 and fold changes > 10% were considered. According to our hypothesis we expected to see distinct patterns in kinase activity between grade I and grades II and III meningiomas. We observed dramatic decrease in all 60 differentially phosphorylated peptides identified in grade III vs I (Supplementary Table S5; Fig. 3c). Conversely, of 34 differentially phosphorylated peptides identified in grade II vs I, 29 were hyperphosphorylated (Supplementary Table S5; Fig. 3c). Like the comparison between grade III and I meningiomas, all but one of the 58 peptides identified in sh33-AKAP12 cells compared to controls was hypophosphorylated. These results indicated that low AKAP12 expression correlate with broadly decreased Ser/Thr kinase activity in Grade III meningiomas and in benign cell line after AKAP12 knockdown. The frequent and discordant increase in kinase activity in atypical meningiomas also suggests that AKAP12 may regulate kinase activity through mechanisms unique to grade III tumors.

### AKAP12 regulates grade II and III meningioma kinome signatures

**Figure 4:**
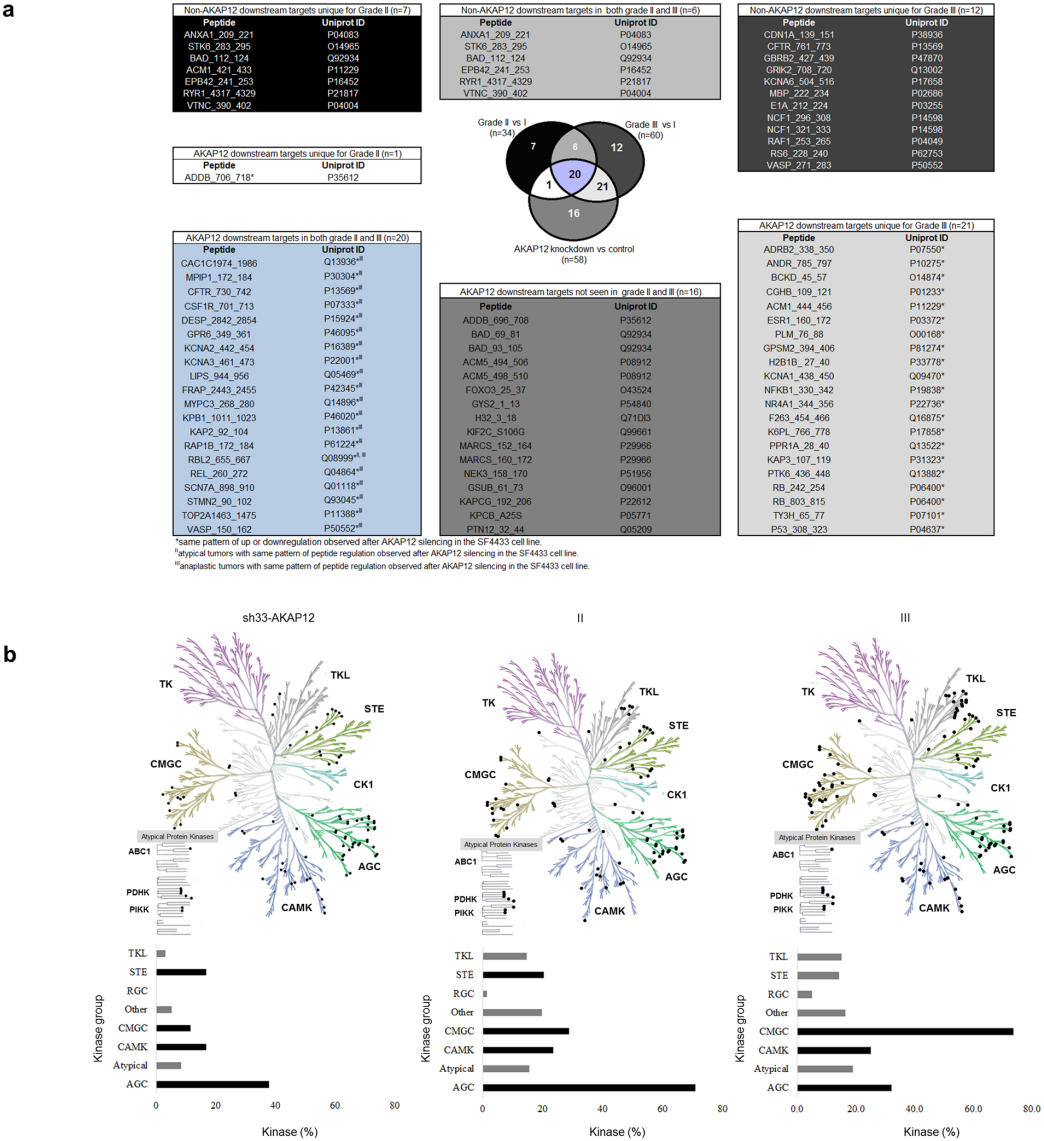
AKAP12 kinome signature in grade II and III meningiomas. (**a**) AKAP12 downstream targets were dysregulated in 62% (21/34) and 68.4% (41/60) of grade II and III meningiomas, respectively. Among these, in grade II and III, 9.52% (2/21) and 100% (41/41) of phosphopeptides, respectively, presented the same dysregulation pattern observed after AKAP12 silencing. These peptides are indicated in the Venn diagram by superscript II and/or III when observed for atypical and/or anaplastic tumors, respectively. In grade IIs, 38.23% phosphopeptides (13/34) did not seem to be AKAP12 downstream targets. In grade IIIs, 30.00% (18/60) phosphopeptides did not seem to be AKAP12 downstream targets. (**b**) Kinases on the phylogenetic tree and bar graphs showing percent of kinases (X axis) classified in each major kinase group (Y axis). Black bars represent AKAP12 regulation of the four major kinase groups: AGC (39.9%) CAMK (16.8%), STE (16.8%), and CMGC (11.6%) which are also overrepresented in in high-grade meningiomas (grade II: AGC 70.8%, CMGC 28.8%, CAMK 23.44, and STE 20.3%; grade III: CMGC 73.6%, AGC 32.1%, and CAMK 25.0%). Kinase tree images are courtesy of Cell Signaling Technology.

To understand whether AKAP12 function is associated with regulation of specific kinase pathways we first assessed the overlap of differentially phosphorylated peptides found in grade II and III meningiomas versus grade I and those differentially regulated by AKAP12 knockdown. This analysis demonstrated regulated peptides unique to grade II (7/34; 21%), III (12/60; 20%) and in sh33-AKAP12 cells (16/58; 28%) while 20 peptides were common to all groups. Nearly 62.0% (21/34) and 68.5% (41/60) of AKAP12 downstream targets were found dysregulated in meningioma grade II and III, respectively. Among these, 9.52% (2/21 in grade II) and 100% (41/41 in grade III) presented same pattern of regulation observed after AKAP12 silencing (Fig. 4a). These data indicated a high degree of overlap among phosphorylated peptides in grade III and sh33-AKAP12 cells and suggests that AKAP12 regulated kinase activity may be similar in anaplastic meningioma and sh33-AKAP12 cells. By contrast, the lack of congruence between phosphorylation states (hyper- or hypophosphorylated) of peptides in grade II and sh33-AKAP12 cells suggests that in grade II meningiomas AKAP12 is influencing shared pathways through upregulating rather than inhibiting kinase activity.

### AKAP12 regulates AGC, MGC, and STE kinase groups in high-grade meningioma

To predict potential kinases involved in the AKAP12 differential phosphorylation, computational analysis was performed using the kinomic data from AKAP12 and meningioma samples. After clustering kinases by groups, we found that AKAP12 knockdown resulted in regulation of four major groups: AGC (39.9%) CAMK (16.8%), STE (16.8%), and CMGC (11.6%) which are the most affected groups in high-grade meningiomas (grade II: AGC 70.8%, CMGC 28.8%, CAMK 23.44%, and STE 20.3%; grade III: CMGC 73.6%, AGC 32.1%, and CAMK 25.0%) (Fig. 4b), strengthen the involvement of the AKAP12 in meningioma malignancy.

### Integrated analysis of global phosphoproteome and kinome enables interpretation of aggressiveness-related networks in high-grade meningioma

**Figure 5:**
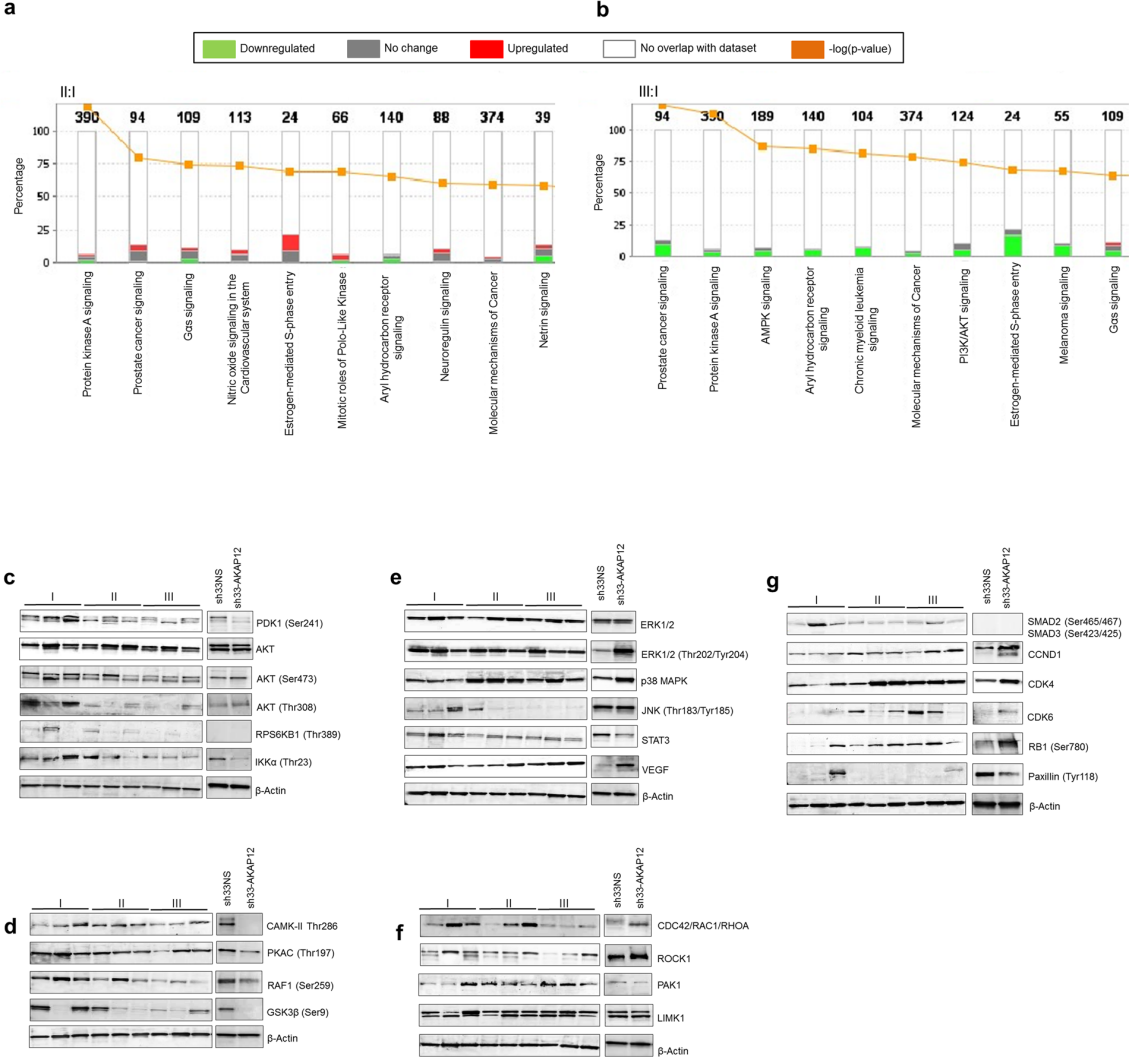
*In silico* analysis and validation of aggressiveness-related kinases in high-grade meningioma. (**a**) Top dysregulated canonical pathways in grade II:I meningioma. (**b**) Top dysregulated canonical pathways in grade III:I meningioma. (**c–f**, and **g**) Western Blots (cropped images) of grade I, II and III tissue lysates, sh33NS and sh33-AKAP12 cell lysates (**c**) PI3K/AKT signaling antibodies (**d**) cAMP/PKA signaling antibodies (**e**) MAPK signaling antibodies (**f**) CDC42-RAC1-RHOA signaling antibodies (**g**) G1/S checkpoint signaling antibodies. See also Supplementary Figures S4, S5, and S6 for Western Blot band quantification.

To determine how AKAP12 and meningioma grade influence global signaling we employed Ingenuity Pathway Analysis (IPA) of STK peptide array (sh33-AKAP12: sh33NS) and the grade II:I and III:I phosphoproteome/kinome combined datasets (iTRAQ LC MS/MS and STK peptide array). For grade II, the PKA, prostate cancer, and Gαs signaling pathways are the most represented cascades (Fig. 5a; Supplementary Table S6). In anaplastic tumors, prostate cancer, PKA, and AMPK cascades were among the ranked cascades (Fig. 5b, Supplementary Table S7). cAMP/PKA, ERK-MAPK, PI3K-AKT and CDC42-RAC1-RHOA are nodes of most of these pathways. The AKAP12 core analysis is shown in the Supplementary Table S8. The highest number of molecules in this data was part of the PKA cascade, followed by mechanisms of cancer, AMPK, GPCR, Insulin, MEK-ERK, and Synaptic Long Term Potentiation (SLTP) (Supplementary Figure S3A). AKAP12 knockdown demonstrated downregulation of molecules involved in the PKA cascade (Supplementary Figure S3B). The PKA pathway was also downregulated in grade II and III datasets (Supplementary Figure S3C and D, respectively). Downregulation of AKT signaling was seen across meningioma grades. AKAP12 seems to downregulate the AKT pathway by inactivating PDK1 Ser241, and IKKα Thr23 in meningiomas (Fig. 5c and Supplementary Figure S4A). Inactivation of the PKA pathway in aggressive meningiomas is also seen despite possible cAMP activation (Fig. 5d and Supplementary Figure S4B) and that AKAP12 silencing downregulates PKA signaling in meningioma. AKAP12 knockdown decreases phosphorylation of JNK Thr183/Tyr185 and STAT3 and increases p38 MAPK, ERK1/2 Thr202/Tyr204, and slightly VEGF. Although phospho-ERK1/2 did not show drastic changes among grades, it is still present at high levels in aggressive tissue samples suggesting that downregulation of AKAP12 may maintain phospho-ERK1/2 in these tumors (Fig. 5e, Supplementary Figure S5A). AKAP12 knockdown increases the expression of CDC42-RAC1-RHOA and ROCK1. Levels of CDC42-RAC1-RHOA, and ROCK1 seems to be more accentuated in atypicals when compared to anaplastics, nonetheless without statistical significance. (Fig. 5f, Supplementary Figure S5B).

### AKAP12 and pathway inactivation confers anaplastic phosphoproteomic profile

The kinome of atypical meningiomas reveals 300% increase in the phosphorylation of the RB1_774_786 peptide. The Western Blotting analysis showed downregulation of SMAD2 Ser465/467/SMAD3 Ser423/425 and upregulation of CCND1, CDK4, CDK6, and RB1 Ser780. AKAP12 knockdown augments the expression in all four targets with a RB1 S780 increase of nearly 500%, 5.0 fold (Fig. 5g, Supplementary Figure S6). The data obtained from the analysis of grade II:I and III:I tumors (combined iTRAQ LC MS/MS and STK peptide array) as well as sh33-AKAP12:sh33NS (STK peptide chip array) was overlaid with PKA (Supplementary Figure 7) and Cell Cycle G1/S Check Point (Supplementary Figure 8) signaling pathways using the IPA software. After AKAP12 silencing, the benign SF4433 cells acquired a similar proteomic profile to anaplastic meningiomas, suggesting that AKAP12 may play a role in meningioma progression.

### AKAP12 expression in a larger and independent cohort of patients by TMA

**Figure 6:**
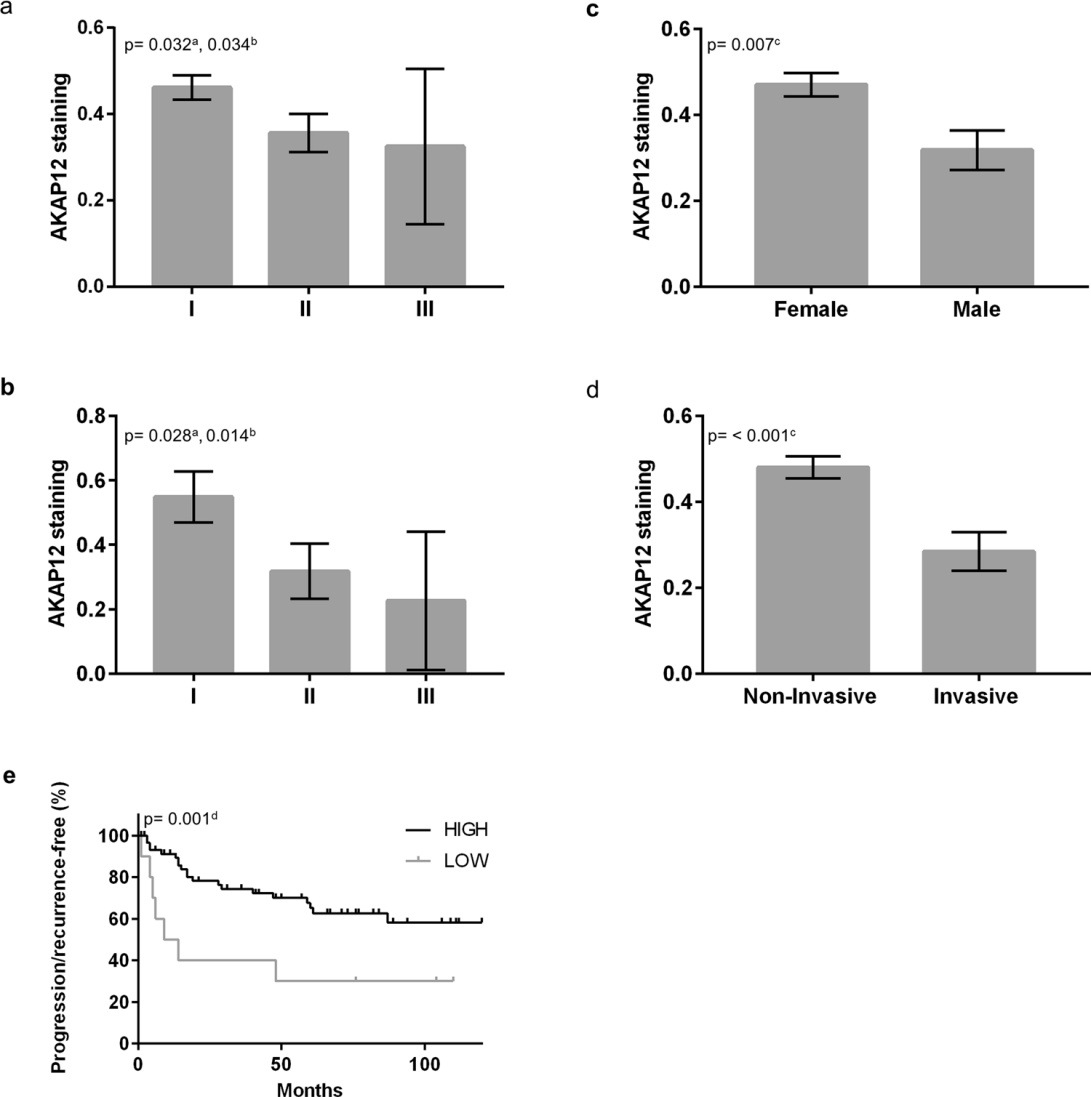
Meningioma anti-AKAP12 staining. (**a**) AKAP12 staining versus tumor grade, all samples (p = 0.032^a^, p = 0.034^b^). (**b**) AKAP12 staining versus tumor grade, samples with prior radiation only (p = 0.028^a^, p = 0.014^b^). (**c**) AKAP12 staining versus patient’s gender (p = 0.007^c^). (**d**) AKAP12 staining in non-invasive and invasive tumors (p = < 0.001^c^). (**e**) Kaplan Meier curve of AKAP12 staining versus percentage of recurrence/progression-free survival independent of tumor grade within 120 months (p = 0.001^d^). ^a^Spearman Correlation. ^b^Rank-regression. ^c^Krustal-Wallis and Spearman correlation as appropriate. ^d^Cox regression. Error bars denote SEM.

In order to investigate the AKAP12 potential as a predictive prognosis marker, we performed the analysis of the AKAP12 expression by Tissue Microarray (TMA) on 81 clinical specimens (Supplementary Table S9). Due to sample limitations, only 75 cores were used for data analysis and interpretation (grade I: 45, grade II: 26, grade III: 4). The AKAP12 expression in different meningioma grades revealed lower AKAP12 expression in high-grade specimens (one-sided Spearman correlation p = 0.032, one-sided rank-regression p = 0.034, Fig. 6a, Supplementary Table S10). An accentuated decrease in AKAP12 staining was verified for high-grade tumors with prior radiotherapy (one-sided Spearman correlation p = 0.028, one-sided rank-regression p = 0.014, Fig. 6b, Supplementary Table S10). The analysis of ancillary effects, such as array position, did not impact AKAP12 staining (Supplementary Table S11). We next correlated relative AKAP12 expression with patient variables (Supplementary Table S11). AKAP12 differs significantly between males and females (p = 0.007), with lower levels in tumors from males (Fig. 6c, Supplementary Table S11). Interestingly, while benign meningiomas are known to show nearly a 2:1 female-male predilection, grade II and III meningiomas demonstrate a male predominance. Tumors with invasive potential show lower AKAP12 staining when compared to non-invasive samples (p =< 0.001) (Fig. 6d, Supplementary Table S11). AKAP12 staining was classified as high: >0.10 or low: <0.10 and analyzed according to the percentage of recurrence/progression-free survival within 120 months. Kaplan Meier curve shows that low AKAP12 values correlated with tumor recurrence/progression (p = 0.001) (Fig. 6e), independent of WHO grade. Low AKAP12 staining was predictive of recurrence, progression and invasion.

## Discussion

**Figure 7:**
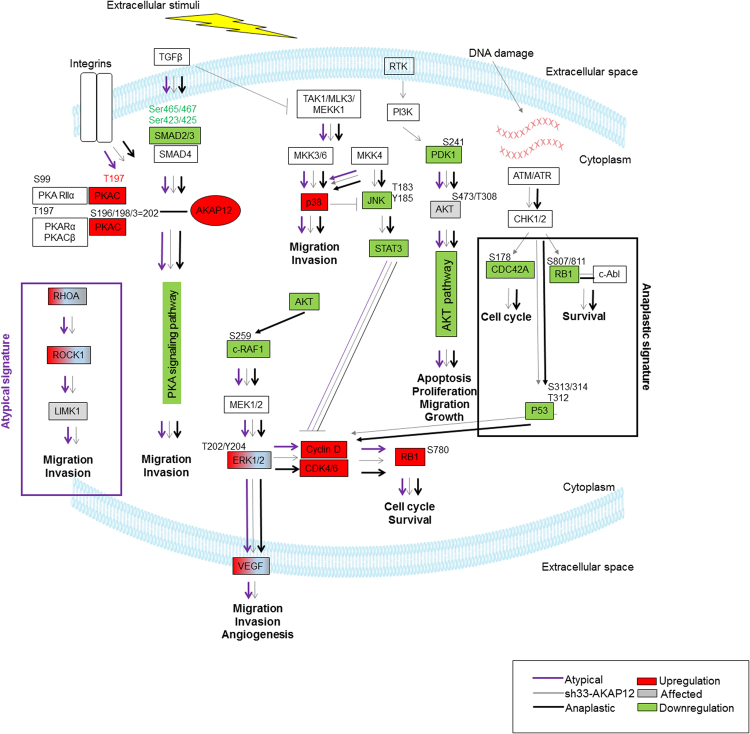
Proposed AKAP12 mechanism to mediate meningioma aggressiveness: Gene names are shown at the approximate positions where their encoded proteins function in the pathway. Although the general organization of the pathway is correct, some details are omitted. Colored molecules were identified experimentally. Grey molecules: affected with no significant changes in regulation. Red molecules: upregulation. Green molecules: downregulation. Purple lines: proposed mechanism in atypical tumors. Black lines: proposed mechanism anaplastic tumors. Grey lines: proposed AKAP12 mechanism in meningioma. Purple and black thick lined boxes indicate atypical and anaplastic signatures, respectively.

We focused on the investigation of high-grade meningiomas since these tumors lack efficient therapies for their management. Drugs targeting *SMO* or *AKT1*mutations^6,7^ in meningiomas are being studied. The clinical importance of mutations in *SMO*, *KLF4*, *TRAF7*, *NF2*, and *AKT1* E17K^6,7^ is unknown. In our meninigioma samples these recurrent variants were not able to predict aggressiveness. Alterations in gene expression, pathways activation, proliferation, cell division or death have been recognized as hallmarks of cancer aggressiveness. We applied high-throughput techniques to profile high-grade meningiomas. AKAP12 in its phosphorylated and unphosphorylated state was decreased in high-grade meningioma tissue and cell lines. We silenced AKAP12 expression in benign meningioma cells, leading to dysregulation of cell cycle, proliferation, migration and invasion. Our results demonstrate that AKAP12 functions as a tumor suppressor in meningioma and regulates cell cycle, influencing cell proliferation, migration and invasion. Since kinase inhibitors have played an important role in cancer treatment, we also explored kinase activity regulated by the AKAP12 in high-grade meningiomas. Simultaneous kinome profiling after AKAP12 silencing and high-grade meningiomas showed a 60% overlap, demonstrating potential for identification of prognostic biomarkers and therapeutic targets. Integration of the phosphoproteome and kinome datasets confirmed the function of AKAP12 in high-grade meningioma signaling. Particularly, the PKA pathway plays a role in high-grade meningiomas. AKAP12 is expressed in low levels in these higher-grade tumors and when it is silenced in meningioma cells, a decrease in PKA signaling results. Interestingly, NF2 has been reported as a direct target of PKA^23^ evidencing the importance of PKA signaling in meningioma aggressiveness. AKAP12 anchors inactive PKA assembling it with a determined set of transduction molecules as well as with members of different pathways^24^. We suggest that low AKAP12 disrupts PKA scaffolding, inactivating downstream signaling, resulting in uncontrolled migration and invasion in aggressive meningiomas (Fig. 7).

The pathway analysis also revealed dysregulation of MAPK. Since AKAP12 downregulation may fail to scaffold PKA resulting in the inhibition of the PKA cascade, it activates ERK1/2 through direct PKC activation and leads to enhanced cellular migration^25,26^ in high-grade meningiomas (Fig. 7). Our results demonstrate hyperphosphorylation of RB1 Ser780 and increased CCND1, CDK4, and CDK6 expression in high-grade meningiomas and after AKAP12 knockdown. CCND1 is required for RB1 phosphorylation at Ser780 *in vivo*. While STAT3 downregulates CCND1^27^, ERK signaling promotes its accumulation^28^. Upregulation of ERK1/2 T202/Y204 and downregulation of STAT3 mediated by AKAP12 may be resulting in the overexpression of CCND1 in high-grade meningiomas. CCND1, CDK4, and CDK6 leads to RB1 phosphorylation and its inactivation releasing E2F1, and inducing cell cycle progression in late G1 phase, which activates cell proliferation^29^ (Fig. 7). Our results are in agreement with the demonstration of the inhibition of meningioma cell proliferation by reducing activation of MEK/ERK pathway with Lovastatin^30^. AKAP12 has been reported to prevent RB-mediated cell senescence and oncogenic progression by attenuating RB activation through PKCα/MEK/Id1/p16^Ink4a^ pathway and preventing downregulation of LASTS1/WARTS by PKCδ in mouse embryonic fibroblasts from akap12-null mice^31^, indicating divergent mechanisms in different models. The *AKT1* E17K reported in meningiomas constitutively activates the PI3K-AKT pathway^32^. As a result, clinical studies with AKT1 inhibitors are under way^33^. Our data revealed downregulation of PI3K-AKT across the grades, which is in contrast to signatures found in cancers that are driven by this variant. We found this mutation in WHO I and II meninigiomas that have been cured by surgery alone, adhering to a very bland phenotype and history (unpublished observations). The *AKT1* E17K was reported in WHO I meningioma, rare in WHO II and absent in anaplastics^34,35^. AKAP12 silencing did not change levels of AKT phosphorylation, although it did lower phosphorylation levels of its downstream targets, indicating participation in the downstream inactivation of the AKT pathway (Fig. 7). AKAP12 originally showed decreased levels of phosphorylation on Ser1587 across the meningioma grades in the iTRAQ LC MS/MS dataset. Characterization of Ser1587 has not been reported although the ATR/ATM are predicted to phosphorylate this specific serine. The kinome of both meningioma samples and cell lines identified decreased phosphorylation levels of CDC25A Ser178, TP53 Ser313/314/Thr312 as well as RB1 Ser807/811 which are targets of ATR/ATM in response to DNA damage. RB Ser807/S811 hypophosphorylation during G1/S phase leads to RB1 and c-Abl binding, inhibiting the c-Abl activity^36,37^. Activation of c-Abl by ATM via DNA damage is associated with apoptosis induction, inhibiting Paxillin Tyr118^38,39^, and interacts with RB inhibiting cell death^40^. TP53 hypophosphorylation on Ser313/314/Thr312 in anaplastic tumors and in meningioma cells after AKAP12 silencing suggests it is a downstream target of AKAP12. DNA damage leads to CHK1/2 Ser313/Ser314 phosphorylation to regulate protein function^41,42^. These observations indicate that, in addition to the RB1, AKAP12 may regulate cell growth and survival through ATM/ATR signaling (Fig. 7). The RHOA-ROCK-LIMK cascade is activated by inhibition of the cAMP-PKA cascade promoting migration and invasion^43^. RHOA-ROCK-LIMK kinase activation was seen after AKAP12 knockdown indicating that AKAP12 may regulate RHOA-ROCK-LIMK pathway in grade II meningiomas. Since AKAPs target the action of PKA by acting as scaffold, our results suggests that AKAP12 downregulation fails to bind to PKA and consequently block phosphorylation of RHOA Ser188 preventing PKA-induced inhibition of RHOA-ROCK-LIMK cascade (Fig. 7). Another particular characteristic of atypical meningiomas found in the present work, was phosphorylation of FRAP_2443_2455, DESP_2842_2854, CDC2_154_169, and CA2D1_494_506 peptides, suggesting activation of the MTOR signaling as well as dysregulation in cascades controlling calcium, and cell cycle. The upregulation of p38 MAPK pathway seen accentuated in atypical samples can negatively regulate JNK^44^ explaining its lower levels observed in high-grade tumors. Activation of p38 MAPK, but not NF-κB or JNK, promotes cell migration by inducing MMP expression^45^ as seen in our *in vitro* investigations. The complexity of kinase activity seen in aggressive meningiomas will require further unbiased screening with specific kinase inhibitors.

AKAP12 knockdown induced decrease in downstream kinase activity in benign cells. When comparing the combined data (iTRAQ LC MS/MS and STK chip array) obtained from grade II and III meningiomas to the kinomic profiling of the sh33-AKAP12, the benign cell line acquired a similar signature to grade III meningiomas, suggesting AKAP12 a role in meningioma progression from grade I to higher grades. Although our results indicate AKAP12 downregulation leads to tumor aggressiveness, purposely increasing AKAP12 expression as a therapy should be considered with caution. Previous data demonstrate increased levels of AKAP12 in cisplatin^46^ and paclitaxel resistant cancer cells^47^. Cisplatin, paclitaxel, and radiation induce DNA breaks, suggesting a cancer protective role for AKAP12. We have observed higher AKAP12 levels in irradiated meningioma cell lines (unpublished observations). Further investigations are required to understand the divergent function of AKAP12 in oncogenesis. Finally, low levels of AKAP12 in an independent cohort was indicative of high-grade, invasive, and recurrent/progressed meningiomas, demonstrating the AKAP12 as a prognosis biomarker.

## Methods

### Patient and tissue selection

Patient data and specimen collection were reviewed and approved by the University of Washington Institutional Review Board and Human Subjects Division. Informed consent was obtained from all subjects. Methods were carried out in accordance with relevant guidelines and regulations.

Patients underwent surgery at the University of Washington Hospitals between January 1^st^ 1998 and December 31^st^ 2012. Data was collected regarding history, demographics, imaging, neuropathology reports, operative information, and outcomes. Resections were re-graded according to revised criteria^2^. Tumors were classified regarding histological subtype, mitoses, Ki-67/MIB, sheeting, macronuclei, hypercellularity, and necrosis. Invasion in operative/pathology reports were coded as invasive. Specimens were reviewed by three neuropathologists. Clinical assessments were performed by neurosurgeons. Total resection was defined as absence of residual enhancement on post-operative MRI within 48 hours of surgery. Recurrence was defined as at least 1 cm of enhancement on subsequent MRI. Progression was considered to be at least 1 cm of growth of a residual tumor on MRI after surgery. The initial discovery set included fourteen meningioma fresh-frozen tissue samples (I:5, II:5, III:4) (Supplementary Table S1). TMA included eighty-one samples (I:50, II:27, III:4) (Supplementary Table S9).

### MIP

DNA was extracted by QIAamp DNA Kit (Qiagen Science, Valencia, CA) and submitted for mutation screening on *SMO*, *KLF4*, *TRAF7*, *NF2*, and *AKT1* E17K point mutation according to O’Roak *et al*.^48^ with modifications. Probes were synthesized by Integrated DNA Technologies (Coralville, IA). Reactions consisting of template (100 ηg), MIPs (5 amoles each), 1x FailSafe PCR 2x PreMix B (Epicentre, Madison, WI), 1x NAD^+^ (New England Biolabs, Ipswich, MA, USA), blocking oligo (10 pmoles), Ampligase (1U) (Epicentre, Madison, WI), FailSafe (0.5U) (Epicentre, Madison, WI) and 4 mM MgCl_2_ were assembled and incubated at 98 °C 5 minutes; 56 °C 5 minutes, and 60 °C 4 hours. Exonuclease I/III (2 µL) were added and incubated at 37 °C 15 minutes and 95 °C 2 minutes. Captured material (5 µL), 2x KAPA Library Amplification Mix (12.5 µL) (KAPA Biosystems, Wilmington, MA), SLXA_PE_MIPBC_FOR and SLXA_PE_MIPBC2_REV (0.5uM each) (57) and water (6.5 µL) were assembled and incubated at 98 °C 30 s; followed by 25 cycles of 98 °C 10 s; 60 °C30s; 72 °C 30s, with final extension at 72 °C 5 minutes. A SLXA_PE_MIPBC2_REV primer, containing a unique 9-base index was used for each template allowing for up to 384 templates to be sequenced as a pool. Reactions were purified and pooled. Reads were mapped to GRCh37 using BWA-MEM^49^. Indel realignment and base quality recalibration was performed^50^. Variants were called using GATK’s HaplotypeCaller in gvcf format and GATK’s GenotypeGVCF.

### Protein extraction

Proteins were extracted from tissue or cell culture in T-PER and M-PER buffers (Thermo Scientific Pierce, Pittsburgh, PA), respectively, supplemented with Halt Phosphatase Inhibitor Cocktail and Halt Protease Inhibitor Cocktail (Thermo Scientific Pierce, Pittsburgh, PA).

### iTRAQ and phosphopeptide enrichment

Equal amounts of protein from individual fresh-frozen tissue of same meningioma grade were pooled creating three groups: WHO I, II, and III. iTRAQ labeling and phosphopeptide enrichment were performed as described previously^51^ with modifications. Pooled lysates were precipitated with acetone, trypsin digested, labeled with tags (Life Technologies, Grand Island, NY; WHO I – iTRAQ114, WHO II – iTRAQ116, and WHO III – iTRAQ117), combined, and desalted. Phosphopeptides were allowed to bind to TiO_2_ spin tips using Phosphopeptide Enrichment and Clean-up Kit (Thermo Scientific Pierce, Pittsburgh, PA), eluted, and cleaned using graphite columns (Thermo Scientific Pierce, Pittsburgh, PA). Samples were dried and resuspended in TFA. LC MS/MS analysis was performed as described previously^51^. The spotted sample plates were analyzed using 4800 Plus MALDI TOF/TOF TM (AB SCIEX, Framingham, MA) with mass range of 800–3500 m/z and S/N > 50. MS/MS spectral data were analyzed using ProteinPilot 4.0 (AB SCIEX, Framingham, MA) referencing International Protein Index (IPI) and UniProtKB/Swiss-Prot database using Proteome Discoverer 1.3 (Thermo Scientific, Pittsburgh, PA) with the following parameters: MMTS for cysteine alkylation, up to two trypsin missed cleavages; biological modification, amino acid and substitutions were set for ID focus; phosphorylation emphasis, FDR < 5%, and protein confidence > 95%. Data was normalized and iTRAQ quantification was expressed as ratio with the WHO I levels (iTRAQ 114) as the denominator. In order to identify relevant candidates in iTRAQ studies, statistical analysis was performed with ProteinPilot on protein ratios based on the weighted average of log ratios and the standard error. In addition, a protein was considered differentially expressed when iTRAQ ratio (WHO II:WHO I, WHO III:WHO I) was ≥ 1.20 or ≤ 0.83 (fold change > 20%). The fold-change cutoff for up- or down-regulation was determined based on pilot studies evaluating the label-specific experimental variation between two replicates for the same experimental group. Similar approaches have been employed by us^51–53^ and others^54,55^ to identify biologically relevant candidates in iTRAQ studies. Proteins were selected for validation based on fold changes, and significance on protein the levels. The spectra of the selected candidates was manually checked to ensure proper site localization.

### STK profiling

The PamStation®12 workstation and STK PamChip® peptide array (PamGene International BV, Hertogenbosch, Netherlands) were used for STK profiling. The fluorescent platform measures the ability of active kinases in a specimen to phosphorylate specific peptides imprinted on multiplex chip arrays. Each chip contains 4 arrays. Each array displays 140 Ser/Thr and 4 positive controls immobilized peptides. Each peptide represents a 15 amino-acid sequence from putative phosphorylation sites in human proteins derived from the literature and correlated with one or multiple upstream kinases. Kinase(s) in the sample actively phosphorylate substrates on the PamChip®, in the presence of ATP. An antibody is used to detect phosphorylated Ser/Thr, and a 2^nd^ FITC-conjugated antibody is used to quantify the phosphorylation signal. Three temperature controlled peptide chips ran in parallel in the PamStation®12. Chips were blocked with 2% BSA (Sigma-aldrich, St. Louis, MO), and each pooled lysate (1 µg) was applied to individual arrays with kinase buffer, 400 µM ATP, and FITC-conjugated antibodies. Signal intensities were quantitated by BioNavigator 6.1.42 (PamGene), expressed per 100 ms exposure and log transformed. Mean value <20% for peptides with a signal >2000 was considered to ensure quality standards. Normalization was applied. Three replicated quantitations were combined using FDR <1%. P-value < 0.05 and >10% fold change were considered significant.

### Western blot

Protein extracts (30 µg) resolved by polyacrylamide gels were transferred to PVDF membranes in 25 mM Tris, 192 mM glycine, 20% methanol, 0.025% SDS, and washed with 1x Tris-buffered saline, 0.1% Tween 20 (1x TBST). After blocking with 3% BSA in 1x TBST, membranes were incubated with primary antibody at 4 °C, overnight. After washing, membranes were incubated with HRP-conjugated secondary antibody. Membranes were developed with Clarity Western ECL Substrate (Bio-rad, Hercules, CA) and visualized in GelDoc XR + System (Bio-rad, Hercules, CA). For loading control, membranes were stripped and re-probed with anti-β-actin. Band intensity was quantified using Image Studio Lite Version 5.0 (LI-COR Biosciences, Lincoln, NE). Antibodies are presented in Supplementary Table S12.

### TMA

Samples were fixed in formalin, processed in tissue processor, and embedded in paraffin to produce FFPE blocks. Regions suitable for TMA were selected in duplicate. TMA was sectioned producing 4 µm sections which were placed on positively charged slides. Deparaffinized rehydrated slides were stained with H&E. Antibody optimization was performed using Leica Bond III Fully Automated IHC and ISH Staining System (Leica Bio-Systems, Buffalo Grove, IL). Anti-AKAP12 (1:50,000) was used. The final step included the Bond Polymer Define Detection System (Leica Bio-Systems, Buffalo Grove, IL), containing endogenous peroxidase blocking, secondary antibody, and a streptavidin–biotin detection system. Slides were counterstained with Gill’s Hematoxylin. Controls were included with antibody run. The slides were scanned using NanoZoomer Digital Pathology System (Hamamatsu Photonics, K. K., San Jose, CA). Images were analyzed by Visiopharm (Hoersholm, Denmark). Visiopharm software converted the initial digital imaging into grayscale values using HDAB – DAB with the Chromaticity Red feature subtracted, an H&E with filter of 3 × 3 pixels, and an RGB - G feature.

### Human mNeningioma cell lines and culture

Ben-Men-1^56^ was purchased from DSMZ (Braunschweig, Germany). HBL-52^57^ was purchased from Servive GmbH (Eppelheim, Germany). Benign SF4433 and SF4068, atypical SF6717, and malignant SF3061^58^ and KT21-MG1^59^ were kindly provided by Dr. Gilson Baia (Johns Hopkins University, Baltimore, MD). CH157-MN (unknown grade)^60^ was provided by Dr. G. Yancey Gillespie (University of Alabama, Birmingham, AL). Cells were cultured at 37 °C, 5% CO_2_ in DMEM/F12 supplemented with 10% FBS, 100 U/mL penicillin, and 0.1 mg/mL streptomycin.

### Transfection and cell-based assays

Transfection with pGIPZ Non-Silencing Lentiviral #RHS4346, pGIPZ GAPDH Lentiviral #RHS4371, and human AKAP12 shRNAs (ID V3LHS_302165 mature antisense TCTTCTGTACGTACAAACT (Open Biosytems, GE Dharmacon, Lafayette, CO) was performed using Lipofectamine 2000 (Life Technologies, Grand Island, New York). Cells were selected using puromycin (30 µg/mL), and sorted using BD FACSAria III Cell Sorter (BD Biosciences, San Jose, CA, USA) with a low forward scatter threshold to detect GFP^+^ cells. Sorting was performed at rate of 2500–3000 events/s. Control sorting was used to ensure a greater than 98.5% efficiency. The green fluorescent population of interest was gated based on light scatter and fluorescence and was visualized under fluorescence microscope Nikon TE-300 (Nikon Instruments Inc., Melville, NY, USA). Proliferation was measured using BrdU Kit (Cell Signaling Technology, Danvers, MA), migration and invasion assays were assessed by CytoSelect Cell Migration and Invasion Assays (Cell Biolabs, San Diego, CA). Cell cycle was assayed by Cell Cycle Kit-Red Fluorimetric (Abcam, Cambridge, MA). Red fluorescence was monitored with FACSCanto II using blue laser 488 ηm excitation and PE channel (Ex/Em = 585/542 ηm). Data was analyzed using FlowJo software (FlowJo LLC, Ashland, OR).

### Bioinformatics

The GO and PCA analyses were performed by PANTHER 10.0^61^. Kinase analysis by GPS 2.1^62^ and PhosphoNet Kinexus. Kinome trees were annotated using Kinome Render^63^. IPA (Ingenuity Systems, Redwood City, CA) was used to identify mechanisms, functions, pathways, and predict upstream regulators. Graphs and Kaplan-Meier curves were generated by GraphPad Prism 4 (GraphPad Software Inc, La Jolla, CA).

### Statistics

Means are presented with error bars indicating SD or SEM. Two groups were compared by Student’s t-test. ANOVA was used for analysis of changes across all meningioma grades. Spearman Correlation and Rank-Regression were applied to examine AKAP12 expression on TMA. Correlation between AKAP12 expression and clinical variables was assessed by Spearman and Kruskal-Wallis as appropriate. Kaplan Meier curves were plotted to identify trends of meningioma recurrence/progression-free survival in terms of high (>0.10) or low (<0.10) AKAP12 staining on TMA independently of grade, and compared statistically by Cox regression. Differences were significant when p < 0.05.

### Data availability

Datasets generated and analyzed during this study are available from the corresponding author on request.

## Electronic supplementary material

Supplementary Figures and Supplementary Tables S1, S2 and S10-S12

Supplementary Tables S3-S9
