# Supplementary material for: Kinome and phosphoproteome of high-grade meningiomas reveal AKAP12 as a central regulator of aggressiveness and its possible role in progression
Source: Sci Rep. 2018 Feb 1;8:2098. doi: 10.1038/s41598-018-19308-y (PMC5794791; doi:10.1038/s41598-018-19308-y)
Supplement: Supplementary file 1 — Supplementary Figures and Supplementary Tables S1, S2 and S10-S12 [file 41598_2018_19308_MOESM1_ESM.doc]

**Supplementary Material**

**Kinome and phosphoproteome of high-grade meningiomas reveal AKAP12 as a central regulator of aggressiveness and its possible role in progression.**

Carolina Angelica Parada PhD^1^, Joshua Osbun MD^1^, Sumanpreet Kaur MSc^1^, Youseff Yakkioui MD^1^, Min Shi PhD^2^, Catherine Pan^2^, Tina Busald^1^, Yigit Karasozen MD^1^, Luis Francisco Gonzalez-Cuyar MD^2^, Robert Rostomily MD^1^ Jing Zhang MD PhD^2^, Manuel Ferreira Jr. MD PhD^1^

^1^Departments of Neurosurgery/University of Washington School of Medicine, University of Washington Medical Center, Seattle/WA, 98195, USA

^2^Department of Pathology/University of Washington School of Medicine, Harborview Medical Center, Seattle/WA, 98104, USA

*Corresponding Author: Manuel Ferreira Jr. 1959 NE Pacific Street 98195, Seattle, WA, USA. Phone: 1 206-543-3570. FAX: 1 206-543-8315. [manuelf3@uw.edu](mailto:manuelf3@uw.edu).

**Supplementary Figures**

**Supplementary Figure S1**: **Gene Ontology (GO) annotation and Principal Component Analysis (PCA) of the 42 phosphopeptides identified protein from iTRAQ LC MS/MS experiments.** The predominant classification of the 42 phosphopeptides identified by the iTRAQ LC MS/MS experiment is highlighted in yellow.

**
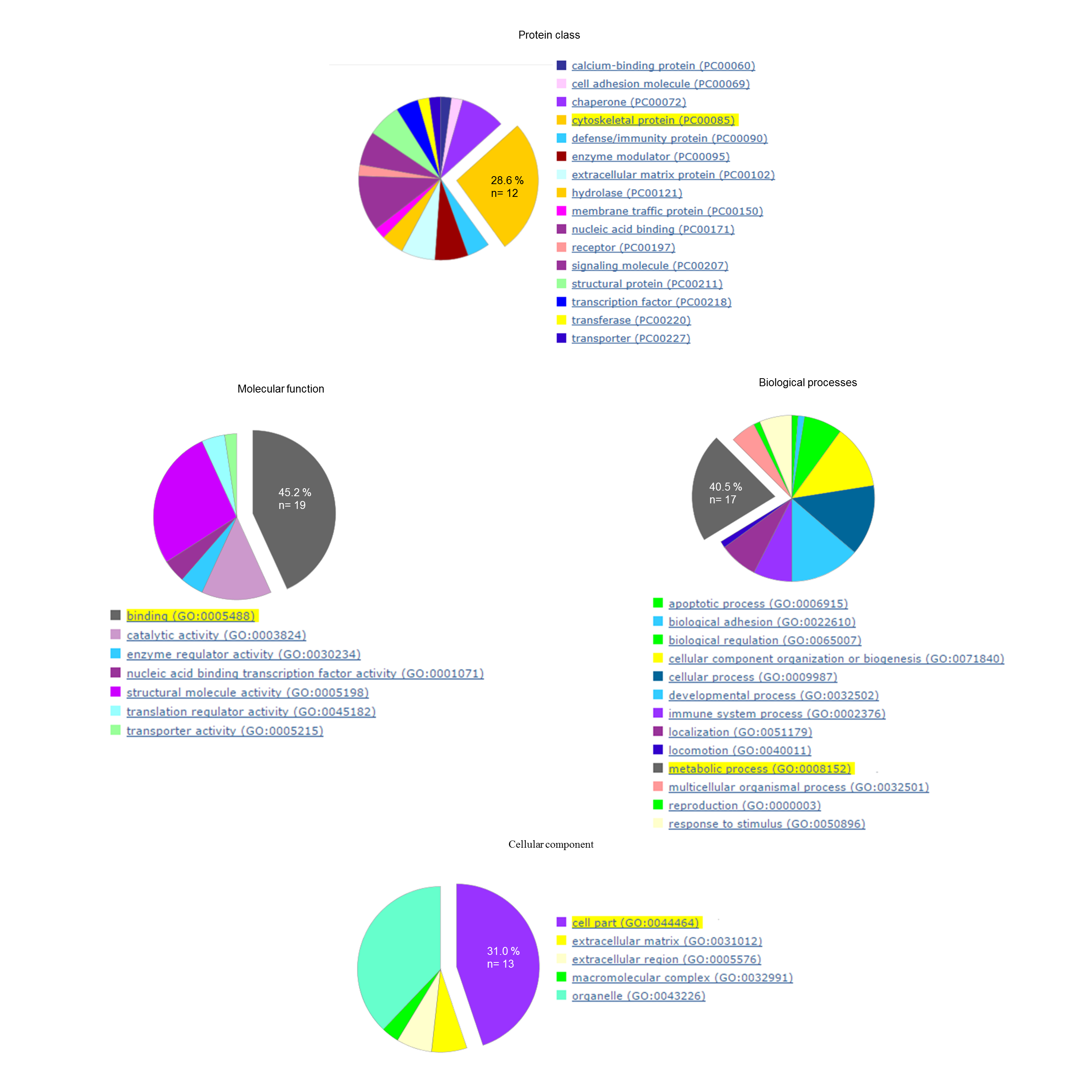
**

**Supplementary Figure S2**: **Statistical analysis of quantification of western blots in Fig. 1C.** Proteins levels were quantitated by densitometry using Image Studio Lite Version 5.0 and normalized to β-ACTIN. Graph represents protein fold (Y xis) versus meningioma grade (X axis). Means are presented with error bars indicating SEM or SD. Two groups (II:I and III:I) were compared by student’s t-test. ANOVA was used for analysis of changes across all three meningioma grades.





**Supplementary Figure S3**: **PKA signaling network downregulation in aggressive meningioma and after AKAP12 gene knockdown**.

**

**

**Supplementary Figure S4**: **Statistical analysis of quantification of western blots in Fig. 5C and D.** Proteins levels or phosphorylation levels were quantitated by densitometry using Image Studio Lite Version 5.0 and normalized to β-ACTIN. Graph represents protein fold (Y xis) versus meningioma grade (X axis). Means are presented with error bars indicating SEM or SD. Two groups (II:I, III:I, and sh33-AKAP12:sh33NS) were compared by student’s t-test. ANOVA was used for analysis of changes across all three meningioma grades.





**Supplementary Figure S5**: **Statistical analysis of quantification of western blots in Fig. 5E and F.** Proteins levels or phosphorylation levels were quantitated by densitometry using Image Studio Lite Version 5.0 and normalized to β-ACTIN. Graph represents protein fold (Y xis) versus meningioma grade (X axis). Means are presented with error bars indicating SEM or SD. Two groups (II:I, III:I, and sh33-AKAP12:sh33NS) were compared by student’s t-test. ANOVA was used for analysis of changes across all three meningioma grades.





**Supplementary Figure S6**: **Statistical analysis of quantification of western blots in Fig. 5G.** Proteins levels or phosphorylation levels were quantitated by densitometry using Image Studio Lite Version 5.0 and normalized to β-ACTIN. Graph represents protein fold (Y xis) versus meningioma grade (X axis). Means are presented with error bars indicating SEM or SD. Two groups (II:I, III:I, and sh33-AKAP12:sh33NS) were compared by student’s t-test. ANOVA was used for analysis of changes across all three meningioma grades.





**Supplementary Figure S7**: A**naplastic phosphoproteomic profile in benign meningioma cell line SF4433 after AKAP12 knockdown.** The data obtained from the analysis of grade II:I and III:I tumors (combined iTRAQ LC MS/MS and STK peptide array) as well as sh33-AKAP12:sh33NS (STK peptide chip array) was overlaid with signaling pathways using the IPA software. **a:** PKA signaling pathway. **b:** Cell Cycle G1/S Check Point. Red: upregulated molecules from the dataset. Green: downregulated molecules from the data set. Grey: affected molecules from the dataset with no significant changes. Dashed arrows = inhibition. Continuous arrows = activation. **
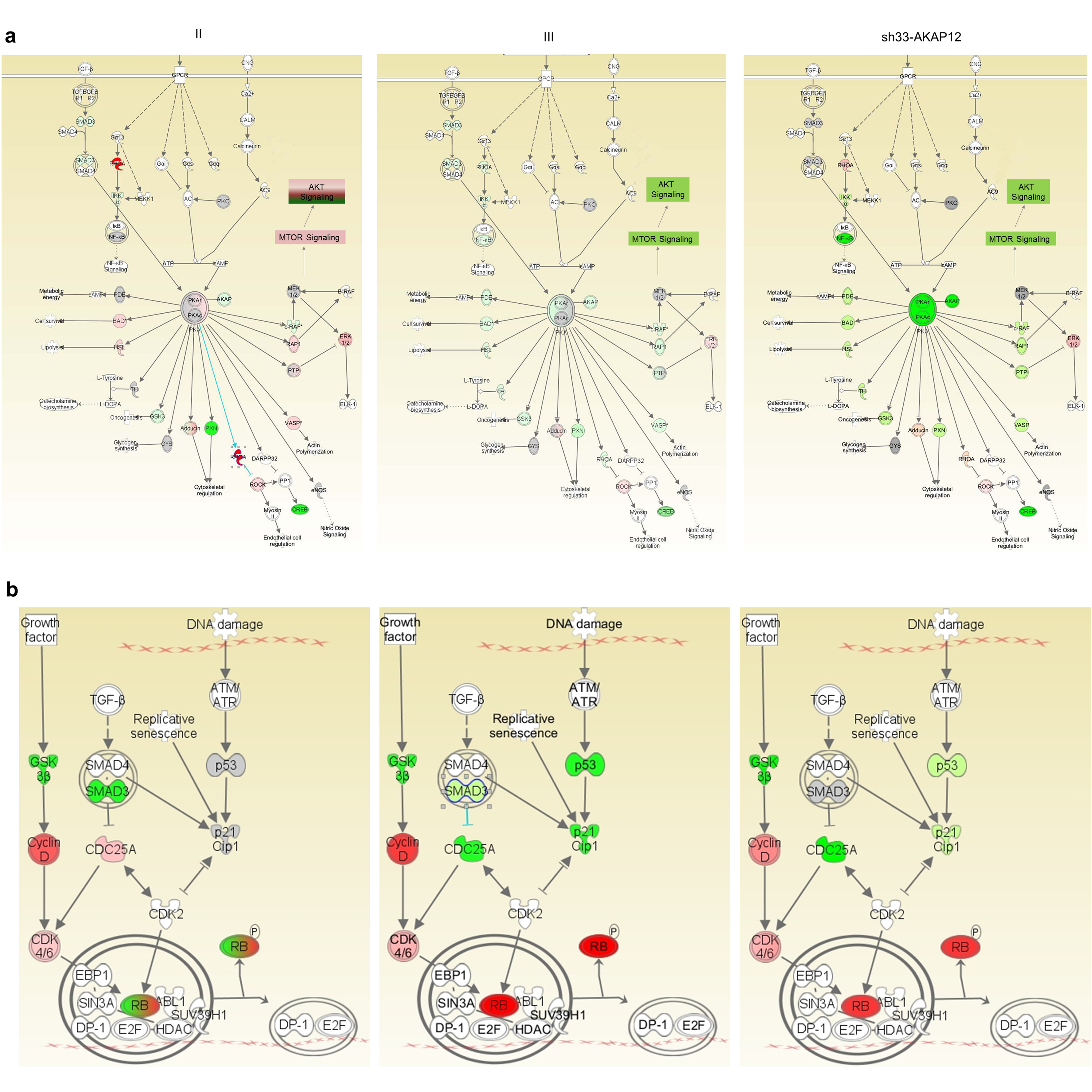
**

**Supplementary Tables**

**Supplementary Table S1: Discovery set of**  meningioma and clinical features.

|  | Patient | Sex | Symptom | Location | Size (cm) | Recurrence (Months) | Previous  Surgeries | Total GK/SRS |
| --- | --- | --- | --- | --- | --- | --- | --- | --- |
| Grade I | 1480 | 59 F | dizziness | R frontoparietal convexity | 3.0 | 0 | 0 | 0 |
|  | 1174 | 50 F | paresthesia  hearing loss | L cerebellar pontine angle | 3.0 | 0 | 0 | 0 |
|  | 1289 | 66 F | behavioral changes  decreased cognitive function decreased memory | falcine frontal | 10.0 x 6.0 | 0 | 0 | 0 |
|  | 1149 | 44 F | seizure | L sphenoid wing | 4.0 x 5.0 | 0 | 0 | 0 |
|  | 1645 | 80 F | gait instability,  weakness  numbness | L frontal | 2.7 x 1.8 | 0 | 0 | 0 |
| Grade II | 1220 | 44 F | headache | R intraventricular | 0.63 x 4.0 | 0 | 0 | 0 |
|  | 1334 | 64F | palpable lump | supratentorial parietal | 4.0 x 2.7 | 67 | 1 | 0 |
|  | 1288 | 72 F | word-finding difficulty  stroke-like episode | L frontotemporal | 0.12 | 5 | 2 | 0 |
|  | 1167 | 40 F | visual changes | R infratentorial | 2.0 | 0 | 0 | 0 |
|  | 1202 | 26 F | headache  visual changes | L parafalcine parietooccipital | 5.0 | 5 | 0 | 0 |
| Grade III | 1109 | 74 F | confusion  weakness | supra and infratentorial | 5.0 x 6.0 | 11 | 0 | 0 |
|  | 1809 | 63 F | headache | bilateral falcine | 6.4 x 5.8 | 12 | 0 | 0 |
|  | 968 | 41 F | headache | R frontoparietal | 5.0 | 0 | 0 | 0 |
|  | 1598 | 65 M | aphasia | L frontotemporal | 4.0 x 3.0 | 9 | 5 | 0 |

F: Female, M: Male, R: Right, L:Left, SRS: Stereotactic radiosurgery, GK: Gamma Knife (GK), Txs: treatment

**Supplementary Table S2: Sequencing for *SMO*, *KLF4*, *TRAF7*, *NF2*, and *AKT* E17K variants by MIP.**

|  | Patient | Type | *NF2* | *TRAF7* | *KLF4* | *SMO* | *AKT*  E17K |
| --- | --- | --- | --- | --- | --- | --- | --- |
| Grade I | 1149 | Tissue |  |  |  |  |  |
|  | 1174 | Tissue |  |  |  |  |  |
|  | 1289 | Tissue |  |  |  |  |  |
|  | 1480 | Tissue | Stop Gained c.794C>A\|p.Ser265 |  |  |  |  |
|  | 1645 | Tissue | Frameshift & Splice Region c.599_600delGG\|p.Arg200fs | Splice Donor c.1086+1G>T |  |  |  |
|  |  |  | Missense c.989C>T\|p.Ala330Val |  |  |  |  |
|  | Ben-Men-1 | Cell line | Frameshift c.639delC\|p.Asp213fs |  |  |  |  |
|  |  |  | LOH |  |  |  |  |
|  | SF4433 | Cell line |  |  |  |  |  |
|  | SF4068 | Cell line |  |  |  |  |  |
|  | HBL-52 | Cell line |  |  |  |  |  |
| Grade II | 1167 | Tissue | Stop Gained c.49A>T|p.Lys17 |  |  |  |  |
|  |  |  | LOH |  |  |  |  |
|  | 1202 | Tissue |  |  |  |  |  |
|  | 1220 | Tissue |  |  |  |  |  |
|  | 1288 | Tissue |  |  |  |  |  |
|  | 1334 | Tissue | Splice Acceptor  c.115-1G>T |  |  |  |  |
|  | SF6717 | Cell line | Frameshift c.949delG\|p.Glu317fs |  |  |  |  |
|  |  |  | Frameshift c.1706delG\|p.Gly569fs |  |  |  |  |
| Grade III | 968 | Tissue | Stop Gained c.1228C>T|p.Gln410 |  |  |  |  |
|  | 1109 | Tissue | Frameshift c.41_42delTC\|p.Leu14fs |  |  |  |  |
|  |  |  | LOH |  |  |  |  |
|  | 1809 | Tissue |  |  |  |  |  |
|  | 1599 | Tissue | Splice Donor  c.599+1G>T |  |  |  |  |
|  |  |  | LOH |  |  |  |  |
|  | SF3061 | Cell line | Frameshift c.1347delA\|p.Glu450fs | Missense  c.1304T>G\|p.Leu435Arg |  |  |  |
|  |  |  |  | Missense  c.1328T>C\|p.Leu443Pro |  |  |  |
|  | KT21-MG1 | Cell line |  | Missense  c.85A>G\|p.Arg29Gly |  |  |  |
|  |  |  |  | LOH |  |  |  |
| Unknown | CH157-MN | Cell line | Splice Acceptor  c.241-1G>T  LOH |  |  | Missense c.1159A>G|p.Ser387Gly |  |
|  |  |  |  |  |  |  |  |

LOH: loss of heterozygosity, del: deletion, fs: frameshift.

**Supplementary Table 10: Statistical analysis of the AKAP12 versus meningioma grade.**

| All samples: with prior and no radiation treatment. | | | | | | | | | |
| --- | --- | --- | --- | --- | --- | --- | --- | --- | --- |
| Marker | Grade | | | Statistical comparisons | | | | | |
|  | I (N=45) | II (N=26) | III (N=4) | Grade II vs I | | Grade III vs I | | Overall | |
|  | Mean %ile | Mean %ile | Mean %ile | Adjusted Difference | Adjusted Significance | Adjusted Difference | Adjusted Significance | **SC Ordinal** | **RR Ordinal** |
| AKAP12 | 55.3 | 41.7 | 44.3 | -10.98 | 0.127 | 7.07 | 0.644 | **0.032** | **0.034** |
|  |  |  |  |  |  |  |  |  |  |
| Irradiated samples only: samples with prior radiation treatment. | | | | | | | | | |
| Marker | Grade | | | Statistical comparisons | | | | | |
|  | I (N=45) | II (N=26) | III (N=4) | Grade II vs I | | Grade III vs I | | Overall | |
|  | Mean %ile | Mean %ile | Mean %ile | Adjusted Difference | Adjusted Significance | Adjusted Difference | Adjusted Significance | **SC Ordinal** | **RR Ordinal** |
| AKAP12 | 71.6 | 38.2 | 32.4 | -35.4 | 0.131 | -10.7 | 0.697 | **0.028** | **0.014** |
|  |  |  |  |  |  |  |  |  |  |

N= number of samples. Statistical significance p<0.05 is highlighted in bold letters. SC = Spearman correlation test (one-sided).

RR = Rank-regression (one-sided significance). %ile: percentile.

**Supplementary Table S11: Univariates.**

| Effect | All grades | | |
| --- | --- | --- | --- |
|  | N (%) | Mean AKAP12 | Significance^a^ |
| Age |  |  |  |
| Mean (SD) | 49 |  |  |
| <40 | 18 (24%) | 0.46 | 0.636 |
| 40-49 | 25 (33%) | 0.38 |  |
| 50+ | 32 (43%) | 0.42 |  |
|  |  |  |  |
| **Sex** |  |  |  |
| **Female** | **49 (65%)** | **0.47** | **0.007** |
| **Male** | **26 (35%)** | **0.32** |  |
|  |  |  |  |
| Hypercellularity |  |  |  |
| No | 58 (77%) | 0.43 | 0.590 |
| Yes | 17 (23%) | 0.38 |  |
|  |  |  |  |
| Necrosis |  |  |  |
| No | 51 (68%) | 0.42 | 0.878 |
| Yes | 24 (32%) | 0.41 |  |
|  |  |  |  |
| Proeminent nuclei |  |  |  |
| Yes | 57 (76%) | 0.45 | 0.063 |
| No | 18 (24%) | 0.31 |  |
|  |  |  |  |
| Sheeting architecture |  |  |  |
| Yes | 56 (75%) | 0.44 | 0.425 |
| No | 19 (25%) | 0.36 |  |
|  |  |  |  |
| Small cell |  |  |  |
| Yes | 74 (99%) | 0.41 | 0.152 |
| No | 1 (1%) | 0.66 |  |
|  |  |  |  |
| **Invasion** |  |  |  |
| **Yes** | **51 (63%)** | **0.48** | **<0.001** |
| **No** | **24 (32%)** | **0.28** |  |
|  |  |  |  |
| Radiation |  |  |  |
| No | 55 (73%) | 0.43 | 0.928 |
| Yes | 20 (27%) | 0.39 |  |
|  |  |  |  |
| Chemotherapy |  |  |  |
| No | 67 (91%) | 0.43 | 0.380 |
| Yes | 7 (9%) | 0.30 |  |
| Unknown | 1 (1%) | 0.59 |  |
|  |  |  |  |
| TMA row |  |  |  |
| A | 10 (13%) | 0.41 | 0.201 |
| B | 8 (11%) | 0.53 |  |
| C | 10 (13%) | 0.48 |  |
| D | 10 (13%) | 0.24 |  |
| E | 7 (9%) | 0.32 |  |
| F | 9 (12%) | 0.45 |  |
| G | 8 (11%) | 0.42 |  |
| H | 10 (13%) | 0.50 |  |
| I | 3 (4%) | 0.38 |  |

Continuation Supplementary Table S11

| Effect | All grades | | |
| --- | --- | --- | --- |
|  | N (%) | Mean AKAP12 | Significance^a^ |
| TMA column |  |  |  |
| 1 | 14 (19%) | 0.45 | 0.492 |
| 2 | 15 (20%) | 0.46 |  |
| 3 | 15 (20%) | 0.42 |  |
| 4 | 16 (21%) | 0.41 |  |
| 5 | 15 (20%) | 0.35 |  |
|  |  |  |  |
| TMA distance from the center |  |  |  |
| 0 | 1 (1%) | 0.47 | 0.137 |
| 1 | 6 (8%) | 0.24 |  |
| 2 | 15 (20%) | 0.38 |  |
| 3 | 19 (25%) | 0.46 |  |
| 4 | 18 (24%) | 0.48 |  |
| 5 | 11 (15%) | 0.34 |  |
| 6 | 5 (7%) | 0.53 |  |
|  |  |  |  |
| TMA Part |  |  |  |
| 1 | 38 (51%) | 0.41 | 0.671 |
| 2 | 37 (49%) | 0.43 |  |
|  |  |  |  |

Statistical significance p<0.05 is highlighted in bold letters.

^a^ Significance by Kruskal-Wallis and Spearman correlation as appropriate.

**Supplementary Table S12: Antibody List.**

| Antibody | Description | Catalogue Number | Vendor | Dilution | |
| --- | --- | --- | --- | --- | --- |
| ADD1 | mouse monoclonal | 54985 | Abcam | 1:1000 | |
| AKAP12 [JP74] | mouse monoclonal | 49849 | Abcam | 1:500 | |
| AKT(pan) [C67E7] | rabbit monoclonal | 4691 | Cell Signaling Technology | 1:1000 | |
| AKT (Ser473) [587F11] | mouse monoclonal | 4051 | Cell Signaling Technology | 1:500 | |
| β-Actin | mouse monoclonal | 8226 | Abcam | 1:3000 | |
| CAMKII [6G9] | mouse monoclonal | 22609 | Abcam | 1:1000 | |
| CAMKII (Thr286) [D21E4] | rabbit monoclonal | 12716 | Cell Signaling Technology | 1:500 | |
| CANX (Ser583) | rabbit polyclonal | 50853 | Abcam | 1:500 | |
| CDC42+RAC1+RHOA | rabbit polyclonal | 170071 | Abcam | 1:500 | |
| CDK4 (D9G3E) | rabbit monoclonal | 12790 | Cell Signaling Technology | 1:1000 | |
| CDK6 (D4S8S) | rabbit monoclonal | 13331 | Cell Signaling Technology | 1:1000 | |
| c-RAF (Ser259) | rabbit polyclonal | 9421 | Cell Signaling Technology | 1:1000 | |
| CREB (Ser133) (87G3) | rabbit monoclonal | 9198 | Cell Signaling Technology | 1:500 | |
| Cyclin D1 (92G2) | rabbit monoclonal | 2978 | Cell Signaling Technology | 1:1000 | |
| GAPDH | rabbit polyclonal | 9485 | Abcam | 1:2500 | |
| GSK3β (Ser9) [D85E12] XP | rabbit monoclocal | 5558 | Cell Signaling Technology | 1:1000 | |
| HSP90B (Ser254) | rabbit polyclonal | 51136 | Abcam | 1:500 | |
| IKKα[3G12] | mouse monoclonal | 11930 | Cell Signaling Technology | 1:1000 | |
| IKKα(Thr23) | rabbit polyclonal | 38515 | Abcam | 1:500 | |
| LIMK1 | mouse monoclonal | 55414 | Abcam | 1:1000 | |
| NUCKS1 | rabbit polyclonal | 77770 | Abcam | 1:250 | |
| p38 MAPK (D13E1) XP® | rabbit monoclonal | 8690 | Cell Signaling Technology | 1:1000 | |
| p44/42 MAPK (ERK1/2) [3A7] | mouse monoclonal | 9107 | Cell Signaling Technology | 1:2000 | |
| Continuation Supplementary Table S12 | | | | |  |
| Antibody | Description | Catalogue Number | Vendor | Dilution | |
| p44/42 MAPK (ERK1/2) (Thr202/Tyr204) [D13.14.4E] | rabbit monoclonal | 4370 | Cell Signaling Technology | 1:1000 | |
| p70 S6K [49D7] | rabbit monoclonal | 2708 | Cell Signaling Technology | 1:1000 | |
| p70 S6K (Thr389) [1A5] | mouse monoclonal | 9206 | Cell Signaling Technology | 1:500 | |
| PAK1 | goat polyclonal | 77096 | Abcam | 1:1000 | |
| PDK1 (Ser241) [C49H2] | rabbit monoclonal | 3438 | Cell Signaling Technology | 1:800 | |
| PGRMC1 | goat polyclonal | 48012 | Abcam | 1:1000 | |
| PKAC (Thr197) | rabbit polyclonal | 4781 | Cell Signaling Technology | 1:500 | |
| RB1 (Ser780) | rabbit polyclonal | 47763 | Abcam | 1:500 | |
| ROCK1 [C8F7] | rabbit monoclonal | 4035 | Cell Signaling Technology | 1:1000 | |
| SAPK/JNK [56G8] | rabbit monoclonal | 9258 | Cell Signaling Technology | 1:1000 | |
| SAPK/JNK (Thr183/Tyr185) [G9] | mouse monoclonal | 9255 | Cell Signaling Technology | 1:500 | |
| SMAD2 (Ser465/467)/SMAD3 (Ser423/425) [D27F4] | rabbit monoclonal | 8828 | Cell Signaling Technology | 1:500 | |
| STAT3 [79D7] | rabbit monoclonal | 4904 | Cell Signaling Technology | 1:1000 | |
| VEGF | rabbit polyclonal | 46154 | Abcam | 1:1000 | |
| Donkey anti-goat IgG H&L (HRP) | secondary anti-goat | 6885 | Abcam | 1:2500 | |
| Mouse IgG HRP-linked | secondary anti-mouse | 7076 | Cell Signaling Technology | 1:2000 | |
| Rabbit IgG HRP-linked | Secondary anti-rabbit | 7074 | Cell Signaling Technology | 1:1000 | |
